# Assessing the ability of GPT-4o to visually recognize medications and provide patient education

**DOI:** 10.1038/s41598-024-78577-y

**Published:** 2024-11-05

**Authors:** Amjad H. Bazzari, Firas H. Bazzari

**Affiliations:** 1 Department of Basic Scientific Sciences, Faculty of Science, Applied Science Private University, Amman, 11931 Jordan; 2 Faculty of Pharmacy, Jerash University, Jerash, 26150 Jordan

**Keywords:** Health services, Patient education, Public health, Therapeutics

## Abstract

Various studies have investigated the ability of ChatGPT (OpenAI) to provide medication information; however, a new promising feature has now been added, which allows visual input and is yet to be evaluated. Here, we aimed to qualitatively assess its ability to visually recognize medications, through medication picture input, and provide patient education via written and visual output. The responses were evaluated by accuracy, precision and clarity using a 4-point Likert-like scale. In regards to handling visual input and providing written responses, GPT-4o was able to recognize all 20 tested medications from packaging pictures, even with blurring, retrieve their active ingredients, identify formulations and dosage forms and provide detailed, yet concise enough, patient education in an almost completely accurate, precise and clear manner with a score of 3.55 ± 0.605 (85%). In contrast, the visual output through GPT-4o generated images illustrating usage instructions contained many errors that would either hinder the effectiveness of the medication or cause direct harm to the patient with a poor score of 1.5 ± 0.577 (16.7%). In conclusion, GPT-4o is capable of identifying medications from pictures and exhibits contrasting patient education performance between written and visual output with very impressive and poor scores, respectively.

**Supplementary Information:**

The online version contains supplementary material available at 10.1038/s41598-024-78577-y.

## Introduction

The use of Chatbots, namely ChatGPT (by OpenAI), has been gaining immense momentum in the past couple of years, reaching over 100 million active users^1^. Numerous studies have illustrated the great capabilities of ChatGPT in dealing with challenging generational tasks and language understanding, highlighting its promising applications among various fields, including medical and healthcare sciences^2,3^.

In May 2024, OpenAI announced the launch of the new flagship model, GPT-4o, with new enhanced features that make it capable of reasoning across audio, vision, and text in real time^4^, a major step-up compared to older models. According to OpenAI, GPT-4o will be able to provide a more natural computer-human interaction, faster response time, and better vision and audio understanding^4^. The aforementioned capabilities can have a significant impact on how chatbots are utilized in daily life. For instance, the addition of visual and audio input can greatly enhance users’ engagement and satisfaction^5^, possibly leading to more reliance on chatbots and their integration in more daily activities, including personal healthcare.

Recent evidence shows significant advances for the accuracy of GPT-4o in various medical fields, including emergency medicine^6^ and dentistry^7^. In addition, many findings indicate impressive visual capabilities; for instance, in radiology examination^8^ and evaluation of the performance of practical skills^9^. Furthermore, ChatGPT exhibits key potentials in healthcare services, especially in relation to patient education, counseling and medications^3^. However, the performance and visual features of GPT-4o are yet to be tested in pharmacy and medication-related services.

Therefore, this study aims to explore and evaluate the new features of GPT-4o in relation to its ability to visually identify medications and provide patient education through text and generated images. As such, the findings of the study would provide further insights into the future applications of artificial intelligence (AI)-powered chatbots in healthcare.

## Methods

The study was conducted through, at the time, the latest version of ChatGPT software (OpenAI, L.L.C., San Francisco, CA, USA), which is known as GPT-4o, and aimed at evaluating its ability to visually recognize medications, through the input of medication package pictures, and provide patient education through both written and visual output. The free version of GPT-4o, which provides the visual input feature, was initially tested; however, it only allowed three inputs every 24 h; thus, the paid version was used for the study. The software was evaluated on the 6th of July 2024.

The interaction was initiated with a simple greeting, followed by a separate input requesting GPT-4o to identify a medication from a picture of its outer packaging and provide information on its indications, proper administration technique, side effects and complications. The input was: “I will show you a picture of a medication I was prescribed, and I want you to tell me what is it, what is it used for, how should I use it, and if there is any potential side effect or complication I should be aware of”. Then, after GPT-4o was allowed to respond, a picture of a medication’s outer packaging was uploaded and the response of GPT-4o was recorded. This process was repeated for a total of 20 medications, which were selected by the authors to cover various pharmacological classes, dosage forms and routes of administration.

The pictures were taken by the authors using a 12-megapixel smartphone camera and compressed, or scaled down, through sharing on WhatsApp application (Meta Platforms, Inc., California, USA). This technique was utilized in order to mimic the most common among the public in terms of: method of capturing pictures, camera resolution and source of picture quality reduction, without necessarily reducing its overall clarity. All responses to the 20 used medication pictures can be found in the supplementary material (Table S1). The list of medications and corresponding properties of pictures uploaded as input to GPT-4o are summarized in Table 1.

**Table 1:** List of used medications and corresponding input picture properties.

No.	Medication	Active Pharmaceutical Ingredient (API)	Dosage Form	API on package?	Picture Size ^1^	Picture Dim ^2^
1	Augmentin^®^	Amoxicillin / Clavulanic acid	Tablets	Yes	61.4	1285
2	Combivent^®^	Ipratropium / Albuterol	Unit Dose Vials	No	57.8	1371
3	Concor^®^ 5 plus	Bisoprolol / HCT ^3^	Tablets	No	60.7	1600
4	Ganaton^®^	Itopride	Tablets	Yes	63.6	1500
5	Lanoxin^®^	Digoxin	Elixir	Yes	54.9	1418
6	Lantus^®^ SoloStar^®^	Insulin glargine	pre-filled pen	Yes	69	1286
7	Lasix^®^	Furosemide	Tablets	Yes	65.1	1465
8	Lyrica^®^	Pregabalin	Hard capsules	Yes	49.8	1500
9	Nexium^®^	Esomeprazole	Tablets	Yes	72.5	1500
10	Omnic Ocas^®^	Tamsulosin	Tablets	Yes	71.7	1500
11	Orfarin^®^	Warfarin	Tablets	Yes	59.4	1456
12	Primolut^®^ Depot	Hydroxyprogesterone	Oily Sol. ampule	Yes	68.9	1282
13	Procto-glyvenol^®^	Tribenoside / Lidocaine	Suppositories	Yes	63.4	1470
14	Rennie^®^	CaCO_3_ / MgCO_3_	Chewable tablets	No	96.6	1500
15	Seretide^®^ MDI	Fluticasone / Salmeterol	MDI ^4^	Yes	72.1	1500
16	Spiriva^®^	Tiotropium	Powder capsules	No	66.6	1500
17	Symbicort^®^ Turbuhaler^®^	Budesonide / Formoterol	Inhalation powder	Yes	52.2	1472
18	Tobrex^®^	Tobramycin	Eye ointment	Yes	54.8	1431
19	Vibrocil^®^	Dimetindene / Phenylephrine	Nasal gel	No	57.2	1459
20	Voltfast^®^	Diclofenac	Powder sachets	Yes	69.7	1374

^1^ Kilobytes, ^2^ Dimensions in pixels (1:1 ratio), 3 Hydrochlorothiazide, 4 Metered-dose inhaler.

In order to assess whether GPT-4o is able to recognize medications through unclear pictures, four medication pictures were blurred and used as input. Two were from the same list: Augmentin^®^ and Seretide^®^, which were moderately and highly blurred, respectively, and two new medications: Crestor^®^ and Glucophage^®^, with moderate and high blurring, respectively.

Next, the ability of GPT-4o to generate responses in the form of visual or image output was assessed in regards to patient education on the proper medication administration technique. Four medications with special administration techniques, other than oral or by mouth, were selected from the list: Lantus^®^ SoloStar^®^ (injection), Seretide^®^ (inhalation), Tobrex^®^ (ophthalmic), and Vibrocil^®^ (intranasal). The following command was used for the first drug: “I was prescribed a medication, I will show it to you in a picture, can you generate an image, or series of images, that shows a step-by-step guide on how to use it?”. Then, the picture of Lantus^®^ SoloStar^®^ medication was uploaded and the generated image was downloaded. The command: “I want you to do the same for this drug” was used for each subsequent medication. Lastly, GPT-4o was prompted to list its sources for the provided medication information in order to assess their reliability: “What were your sources of information regarding the drugs that I have shown you?”.

The responses of GPT-4o were recorded and evaluated based on accuracy, precision and clarity using a 4-point Likert-like scale, which is commonly adopted in the literature as a tool to evaluate large language models in healthcare^10^. The response accuracy was measured based on the correctness of the answer. Precision was assessed by how specific and relevant the response is, and clarity was assessed based on the ease with which a patient could read and understand the provided information. The evaluation criteria for response scoring are detailed in Table 2. Accordingly, a score of 4 indicates “accurate, precise and clear response”; a score of 3 indicates “almost accurate, precise and clear response”; a score of 2 indicates “partially accurate, precise and clear response”; and a score of 1 indicates “inaccurate, imprecise or unclear response”. The scoring of responses was jointly performed by both authors (A.H.B: PharmD, MSc in pharmacology, PhD in pharmacology and translational neuroscience and F.H.B: BPharm, MSc in clinical pharmacology, PhD in pharmacology and toxicology). The score ranks were compared between written and visual responses, and between clear and blurred picture responses, using Mann-Whitney U test and the effect size for the difference in means was assessed using Cohen’s d value.

**Table 2:** Evaluation criteria for scoring GPT-4o’s responses.

Accuracy*		Precision*		Clarity*	
Level	Score	Level	Score	Level	Score
The answer is completely correct and factual without any mistakes	4	The answer directly provides all requested information without irrelevant details	4	The answer is completely clear and understandable	4
The answer is almost completely correct with minor incorrect details	3	The answer provides requested information with few missing details or with some irrelevant details	3	The answer is mostly clear with few ambiguous details	3
The answer is partly correct with some incorrect information	2	The answer misses a part of the question or provides many irrelevant information	2	The answer is partly clear with some confusing parts	2
The response is mostly incorrect or with major factual errors	1	The response is irrelevant to, or doesn’t answer, the questions	1	The response is mostly unclear or incomprehensible	1

* The lowest possible score among the three criteria was used to determine the overall score for each answer.

## Results

GPT-4o complied with the initial request to provide medication information from a picture by responding with “Sure, please go ahead and upload the picture of the medication. I’ll do my best to provide you with the information you’re looking for.”. The responses of GPT-4o to each of the 20 medication picture inputs started with a sentence stating the brand name of the medication, which is found on its packaging, and its active ingredient(s), even for medication pictures in which the active ingredient does not appear, followed by written answers to each of the requested information: “what is it?”, “what is it used for?”, “how should you use it?” and “potential side effects and complications”. All responses ended with a “Note” by GPT-4o to “Always follow your healthcare provider’s instructions closely and report any unusual symptoms or side effects immediately”, followed by additional advice specific to each medication.

Each answer was scored according to its accuracy, precision and clarity using a 4-point Likert-like scale, from 1 to 4 (Table 3). The mean score was 3.55 ± 0.605 indicating almost completely accurate, precise and clear answers. The mean answer word count was 283.25 ± 31.41 words, which translates to an average reading time of around two minutes, indicating relatively concise responses. Of the 20 medication responses, 12 received a score of 4 and 7 received a score of 3 while only one response received a score of 2; therefore, a score of 4 was the median and the mode. None of the written responses to the 20 medication pictures were given a score of 1 and none were labeled as incorrect or considered directly harmful to patients. Accordingly, the point deductions were due to either partial unclarity or, in most cases, the lack of certain administration steps or medication-specific considerations. All written GPT-4o responses to the 20 medication picture inputs can be found in the supplementary material (Table S1).

**Table 3:** Summary of GPT-4o response scores and word count.

No.	Medication	Response score ^1^	Response word count
1	Augmentin^®^	4	316
2	Combivent^®^	4	307
3	Concor^®^ 5 plus	4	302
4	Ganaton^®^	3	227
5	Lanoxin^®^	4	285
6	Lantus^®^ SoloStar^®^	2	254
7	Lasix^®^	3	282
8	Lyrica^®^	3	295
9	Nexium^®^	4	289
10	Omnic Ocas^®^	3	256
11	Orfarin^®^	4	319
12	Primolut^®^ Depot	4	254
13	Procto-glyvenol^®^	4	253
14	Rennie^®^	3	231
15	Seretide^®^ MDI	3	318
16	Spiriva^®^	3	257
17	Symbicort^®^ Turbuhaler^®^	4	302
18	Tobrex^®^	4	315
19	Vibroci^®^	4	269
20	Voltfast^®^	4	334
Mean		3.55	283.25
Standard Deviation		0.605	31.41
95% Confidence Interval		3.27–3.83	268.55–297.95

^1^ Likert-like scale from 1 to 4 (higher scores are more accurate, precise and clear answers).

Next, the ability of GPT-4o to recognize medications from blurred pictures was assessed. Indeed, GPT-4o was able to recognize all four tested medications and provide the requested information irrespective of the degree of blurring or if the medication picture was previously used as input or not. Interestingly, the responses to the pictures of Augmentin^®^ and Seretide^®^, which were used as input beforehand without blurring, were almost identical in terms of provided information to the initial responses to these two medications, indicating a good level of consistency. The mean score obtained for the four blurred medication pictures was 3.75 ± 0.5 with a mean answer word count of 317.5 ± 7.14, which also indicates an almost completely accurate, precise and clear answers with a mean reading duration of around two minutes. The score ranks did not significantly differ between clear and blurred picture responses (*P* > 0.05), and the effect size for the difference in means was small (d = 0.338). The GPT-4o responses to the four blurred medication pictures are provided in the supplementary material (Table S2).

The assessment of GPT-4o’s written responses revealed that it does not only provide the requested information in detail but offers additional relevant considerations and clarifications that could enhance patient understanding and thus the overall patient education outcomes. To mention a few examples: GPT-4o stressed the importance of completing the entire prescribed course of an antibiotic to prevent resistance; advised to take a medication that contains a diuretic in the morning, or in the morning and afternoon if taken twice daily, to avoid needing to micturate during the night; provided dietary advice when educating about a medication prone to drug-food interactions; and stressed that an intramuscular drug is to be administered by a healthcare professional. Other clarifications related to how the patient could identify the occurrence of certain side effects or complications; for instance, it mentioned the signs and symptoms of allergic reactions, infections, hypoglycemia, dehydration, electrolyte imbalance, liver or kidney problems and medication-specific withdrawal symptoms and toxicities. Furthermore, GPT-4o clarified many medical terms that a patient may not be familiar with such as jaundice, bronchospasm, hypoglycemia, orthostatic hypotension, arrythmia, lipodystrophy, subcutaneous and angioedema among many others. In addition, all provided medication dosing regimens, regarding the dose, frequency and duration, were accurate while stressing that these might vary between patients and thus the need to follow the exact physician’s instructions.

**Fig. 1:**
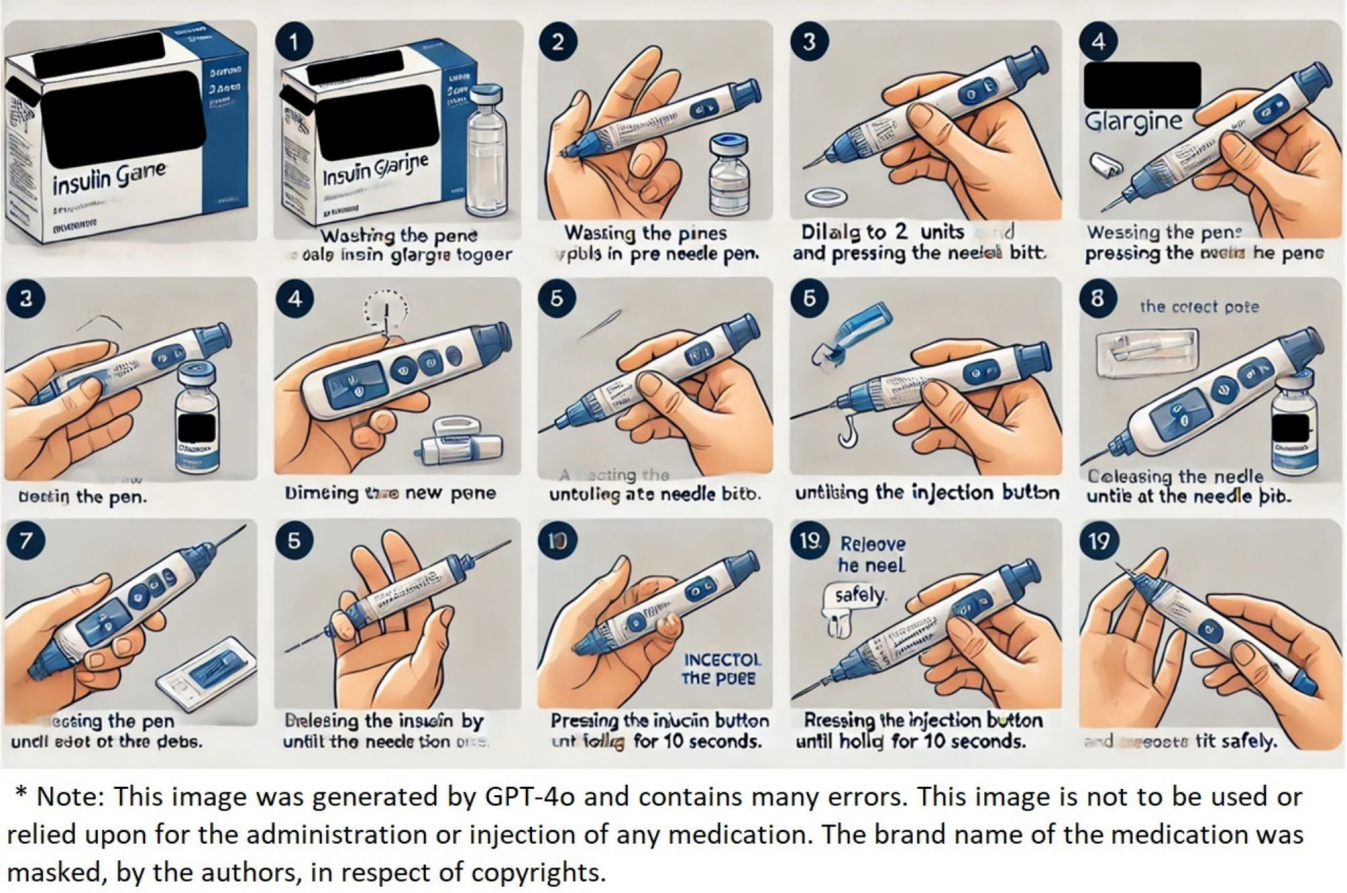
An image generated by GPT-4o to show the administration technique of an injectable medication in a pre-filled pen, containing many errors. The input was a picture of Lantus^®^ SoloStar^®^ medication packaging with a request to generate an image showing how to use it. * The note underneath the image was added by the authors.

The next step was to assess the visual output of GPT-4o regarding the administration technique of certain medications. In contrast to its impressive performance in providing written medication information and usage instructions, the generated images by GPT-4o were unsatisfactory and contained many errors. An example is shown in Fig. 1 for the injection of a pre-filled insulin pen.

GPT-4o responded with “Here is a step-by-step guide to using Lantus^®^ SoloStar^®^ insulin glargine in a pre-filled pen. Each step is illustrated to help you use it correctly. If you have any questions or need further assistance, feel free to ask!” to the first medication input with a similar response to the three subsequent medications. Each of the images generated by GPT-4o was collage-like of multiple smaller images, starting with a drawing of the medication packaging with its brand name and other unclear text, followed by a series of numbered steps illustrating the usage or administration of the medication, each with a caption explaining the corresponding step. However, all generated images had many mistakes and were deemed unusable and potentially harmful. In terms of medication representation, the package drawings were different from the original ones in appearance including the shape and color. In relation to the actual steps of administration, they were unclear and, in many cases, completely incorrect; for instance, showing the injection of insulin in the hand and applying a nasal gel on the face. Regarding the explanatory text underneath many of the steps, it was either unreadable with spelling errors or merged words, or unrelated to the shown step. The numbering of steps was also incorrect with many missing, repeated or unordered correctly. Accordingly, the visual output of GPT-4o regarding medication usage failed in relation to accuracy, precision and clarity with a mean score of 1.5 ± 0.577 (equating to a score of 16.7%). Indeed, the visual output scores were significantly lower, by ranks, compared to the written response scores (*P* < 0.01) with a strong effect size for the difference in score means (d = 3.41). In addition to the image shown in Fig. 1, the GPT-4o-generated images of the three other medications can be found in the supplementary material (Table S3).

Lastly, when GPT-4o was asked to list its sources for drug information it responded by stating various reliable sources of medication information. The initial part of its response was: “The information provided about the medications is based on widely accepted medical knowledge and standard drug reference sources. Specifically, the data is compiled from:

1. **Drug Monographs**: Comprehensive documents that provide detailed information on drugs, including their uses, dosages, side effects, and contraindications.
2. **Pharmaceutical Manufacturers’ Product Information**: Official information provided by the manufacturers of the drugs.
3. **Medical and Pharmaceutical Textbooks**: Authoritative texts used by healthcare professionals to reference drug information.
4. **Clinical Guidelines**: Guidelines provided by health organizations such as the American Medical Association (AMA), World Health Organization (WHO), and others”.

The response of GPT-4o regarding its sources for medication information continued with: ”The specific information sources for each drug include:” and stated the following information sources specific for each of the assessed medications and in the order they were used as input:

- Amoxicillin Trihydrate and Potassium Clavulanate (Augmentin^®^): Product information from GlaxoSmithKline (GSK) and guidelines provided by health organizations such as the American Medical Association (AMA), World Health Organization (WHO), and others.
- Ipratropium Bromide and Albuterol (Combivent^®^): Product information from Boehringer Ingelheim and COPD management guidelines.
- Bisoprolol and Hydrochlorothiazide (Concor^®^ 5 Plus): Product information from Merck and hypertension management guidelines.
- Itopride (Ganaton^®^): Drug monographs and gastrointestinal motility disorder treatment guidelines.
- Digoxin (Lanoxin^®^): Drug monographs and cardiovascular treatment guidelines.
- Insulin Glargine (Lantus^®^ SoloStar^®^): Product information from Sanofi and diabetes management guidelines.
- Furosemide (Lasix^®^): Drug monographs and guidelines for diuretic use in cardiovascular and renal conditions.
- Pregabalin (Lyrica^®^): Product information from Pfizer and neuropathic pain management guidelines.
- Esomeprazole (Nexium^®^): Product information from AstraZeneca and gastroesophageal reflux disease (GERD) treatment guidelines.
- Tamsulosin (Omnic Ocas^®^): Drug monographs and clinical guidelines for BPH treatment.
- Warfarin (Orfarin^®^): Drug monographs and clinical guidelines on anticoagulants.
- Hydroxyprogesterone Caproate (Primolut^®^ Depot): Product information from Bayer and clinical guidelines on hormone treatments.
- Tribenoside and Lidocaine (Procto-Glyvenol^®^): Product information and clinical guidelines for hemorrhoid treatment.
- Calcium Carbonate and Magnesium Carbonate (Rennie^®^): Product information from Bayer and guidelines on antacid use.
- Fluticasone Propionate and Salmeterol Xinafoate (Seretide^®^): Product information from GlaxoSmithKline and respiratory disease treatment guidelines.
- Tiotropium Bromide (Spiriva^®^): Product information from Boehringer Ingelheim and COPD management guidelines.
- Budesonide and Formoterol (Symbicort^®^): Product information from AstraZeneca and asthma/COPD management guidelines.
- Tobramycin (Tobrex^®^): Product information from Alcon and ophthalmic antibiotic use guidelines.
- Dimethindene Maleate and Phenylephrine Hydrochloride (Vibrocil^®^): Product information from Novartis and nasal decongestant use guidelines.
- Diclofenac Potassium (Voltfast^®^): Product information from Novartis and NSAID use guidelines.
- Rosuvastatin (Crestor^®^): Product information from AstraZeneca and guidelines for statin therapy.
- Metformin (Glucophage^®^): Product information from Merck and diabetes management guidelines.”.

## Discussion

This study is the first to provide a qualitative assessment of the newly introduced ability of GPT-4o of receiving visual input and evaluate the quality of generated visual output in regards to the provision of medication information and patient education. The study revealed contrasting performance between the two and identified various corresponding strengths and weaknesses.

In terms of handling visual input and providing written information, GPT-4o was able to recognize all tested medications from their provided packaging pictures, retrieve their active pharmaceutical ingredients, identify the exact formulations and dosage forms of the medications and provide impressively detailed patient education in an almost completely accurate, precise and clear manner. This includes the pharmacological classification; the indications; the use instructions in relation to dosing and administration; the potential side effects and complications; and any other precautions and drug-specific considerations. Other observed advantages include the handling of blurred or unclear pictures, consistency of answers, conciseness of responses, clarification of medical terms, reliability of information sources and emphasis on medication adherence and the importance of consulting and following the exact instructions of healthcare professionals. On the other hand, minor disadvantages were observed such as the limitation of total inputs by the free version of GPT-4o and the missing of few administration steps of some inhaler and injectable medications.

Various studies have investigated the ability of, mainly the previous versions of, ChatGPT in providing medication information and solving pharmacological questions and clinical case, showing highly promising results with good performance^11–14^. However, some other studies showed a weakness of ChatGPT in handling more complex clinical cases requiring advanced reasoning^15^, answering examination questions in certain fields of pharmacy study such as pharmaceutics and biopharmaceutics as opposed to pharmacology^16^, and handling problem-solving multiple-choice questions requiring reasoning^17^. Accordingly, these findings highlight that the performance of ChatGPT can vary based on the type, specialty and complexity of questions. Regarding the visual processing capabilities of ChatGPT, the current results are consistent with previous evidence showing its visual robustness and accuracy in standard^18^ and challenging^19^ images. This is in contrast to its limitations against intentionally deceptive visual inputs^20^ and the complex interpretation of medical images^21^.

The capability of ChatGPT in providing patients with the necessary medication information and solving basic life-like scenario patient questions as we have shown previously^11^, together with the current results showing that a picture of a medication’s packaging is all what a patient may need to obtain all relevant medication information provide compelling evidence for the potential usefulness of ChatGPT application in tele-pharmacy and patient education. However, certain considerations must to be taken into account such as the limitation of allowed inputs on the free version, whether GPT-4o will be able to identify local medication brand names and the potential harm of any inaccurate or missing details, especially on a large-scale application. This would especially be true for cases involving off-label drug uses, special administration techniques or atypical dosing, all of which being patient-specific. Therefore, ChatGPT output should always be approached carefully when answering medication-related questions.

In relation to the visual output of GPT-4o, the results were concerning as the generated images on drug administration contained many errors that would either hinder the effectiveness of the medication or cause direct harm to the patient. The significant difference in performance results between written and visual output suggests that GPT-4o utilizes different mechanisms, and potentially information sources and training data, in this regard. This is supported by the inconsistencies of medication administration instructions between written and image outputs and the almost complete unreadability of text found within the generated illustrations. Accordingly, GPT-4o-generated images should not be relied upon without professional oversight. Another point for consideration is the lack of watermarks or any other indicators on the output images to specify that these are GPT-4o-generated or to emphasize on the consultation of a healthcare professional or referral to the medication’s patient information leaflet. Further developments to the visual output of GPT-4o are warranted before any medical application can be suggested.

The current study has a number of limitations for consideration by future studies: (1) the response evaluation was jointly performed by the authors which hindered the calculation of inter-rater reliability, which is a measure of the agreement level of ratings; (2) the sample size of 20 medications for written output, and 4 medications for visual output, is relatively small and may not be enough for result generalization; and (3) the lack of a control group such as practicing pharmacists, experts or other AI-powered chatbots, which would have allowed a better quantification of how well GPT-4o performed.

## Conclusion

GPT-4o is impressively capable of identifying medications from their outer packaging pictures and retrieving their information. GPT-4o displayed varied patient education performance with satisfactory written output and poor visual output.

## Electronic supplementary material

Below is the link to the electronic supplementary material.

Supplementary Material 1

## Data Availability

All responses of GPT-4o are provided within the manuscript or can be accessed through the supplementary information files: the GPT-4o responses to the 20 medication pictures (Table S1); the GPT-4o responses to the four blurred medication pictures (Table S2); and the GPT-4o-generated images with their corresponding inputs (Table S3). The medication pictures used as input can be obtained from the corresponding author upon reasonable request.
