# Supplementary material for: Assessing the ability of GPT-4o to visually recognize medications and provide patient education
Source: Sci Rep. 2024 Nov 5;14:26749. doi: 10.1038/s41598-024-78577-y (PMC11538418; doi:10.1038/s41598-024-78577-y)
Supplement: Supplementary file 1 — Supplementary Material 1 [file 41598_2024_78577_MOESM1_ESM.pdf]

Supplementary Table S1

No.	Input (medication package picture)	Response (output)
**1**	"I will show you a picture of a medication I was prescribed, and I want you to tell me what is it, what is it used for, how should I use it, and if there is any potential side effect or complication I should be aware of"<br><br>Picture of: Augmentin®	The medication in the picture is Augmentin. Augmentin is a combination antibiotic that contains two active ingredients: amoxicillin and clavulanate potassium. **What is it?** Augmentin is a combination antibiotic used to treat various bacterial infections. It combines amoxicillin, a penicillin-type antibiotic, with clavulanate potassium, which helps prevent certain bacteria from becoming resistant to amoxicillin. **What is it used for?** Augmentin is commonly prescribed to treat infections such as: Sinus infections (sinusitis), Ear infections (otitis media), Lower respiratory tract infections (such as bronchitis and pneumonia), Skin infections, Urinary tract infections (UTIs) **How should I use it?** Dosage and Duration: Always follow your doctor's instructions regarding the dosage and duration of the medication. Typically, it is taken every 8 or 12 hours depending on the specific infection and severity. With Food: It is generally recommended to take Augmentin at the start of a meal to reduce potential gastrointestinal discomfort. Complete the Course: Make sure to complete the entire prescribed course, even if you start to feel better before finishing it, to ensure the infection is fully treated and to prevent antibiotic resistance. **Potential Side Effects or Complications:** While Augmentin is generally well-tolerated, it may cause some side effects. Common side effects include: Nausea or vomiting, Diarrhea, Skin rash, Vaginal yeast infection. Less common but more serious side effects include: Severe allergic reactions (anaphylaxis), which require immediate medical attention, Liver problems, such as jaundice (yellowing of the skin or eyes), Severe skin reactions, Clostridium difficile-associated diarrhea, which can occur during or after treatment. **Precautions**: Allergies: Inform your doctor if you have a history of allergies to penicillin or cephalosporin antibiotics. Medical Conditions: Discuss any other medical conditions you have, such as liver disease or kidney disease, with your doctor. Interactions: Tell your doctor about any other medications you are taking, as Augmentin can interact with other drugs. If you experience any severe side effects or signs of an allergic reaction, seek medical attention immediately. **Note:** Always consult your doctor or pharmacist for more detailed information tailored to your specific situation.
**2**	Picture of: Combivent®	The medication shown in the image is Combivent Unit Dose Vials, which contain a solution for inhalation. Each vial is 2.5 ml. The active ingredients in Combivent are ipratropium bromide and albuterol (salbutamol) sulfate. **What is it?** Combivent is a combination of two bronchodilators: Ipratropium bromide: An anticholinergic that helps to relax the muscles around the airways. Albuterol (salbutamol) sulfate: A beta-agonist that also helps to relax the muscles around the airways. **What is it used for?** Combivent is used to: Treat chronic obstructive pulmonary disease (COPD), including chronic bronchitis and emphysema. Provide relief from bronchospasm (narrowing of the airways) in people with COPD. **How should you use it?** Dosage: Follow your doctor's prescription exactly. Typically, one vial is used in a nebulizer 3-4 times daily. Administration: Use a nebulizer machine to inhale the medication. Empty the contents of one vial into the nebulizer cup. Connect the nebulizer to the air compressor. Sit upright and place the mouthpiece in your mouth or the mask over your nose and mouth. Breathe in the medication deeply and slowly until the nebulizer stops producing mist (usually about 5-15 minutes). Frequency: Use as directed by your healthcare provider, typically 3-4 times daily. **Potential side effects and complications:** Common side effects: Dry mouth or throat. Cough. Nervousness. Tremors. Dizziness. Headache. Nausea. Serious side effects: Severe allergic reactions (rash, itching/swelling, severe dizziness, trouble breathing). Chest pain or palpitations. Increased heart rate. Difficulty urinating. Severe bronchospasm (wheezing, chest tightness, difficulty breathing). **Note:** Always follow your healthcare provider's instructions closely and report any unusual symptoms or side effects immediately. Do not exceed the prescribed dosage, as overuse can lead to serious cardiovascular side effects. If you experience any difficulty breathing or worsening symptoms, seek medical attention immediately. Regular follow-ups with your healthcare provider are important to monitor your condition and the effects of the medication.

**3**	Picture of: Concor® 5 plus	The medication shown in the image is Concor 5 Plus, which contains the active ingredients bisoprolol and hydrochlorothiazide. **What is it?** Concor 5 Plus is a combination medication that includes bisoprolol, a cardioselective beta-blocker, and hydrochlorothiazide, a diuretic (water pill). Bisoprolol works by slowing down the heart rate and reducing the workload on the heart, while hydrochlorothiazide helps reduce fluid retention by increasing urine output. **What is it used for?** Concor 5 Plus is used to: Treat high blood pressure (hypertension). Manage chronic heart failure. **How should you use it?** Dosage: Follow your doctor's prescription exactly. The typical dose is one tablet taken once daily, but the exact dosage will depend on your specific condition and response to the medication. Administration: Take the tablet orally with a glass of water. It can be taken with or without food, preferably in the morning. Consistency: Take it at the same time each day to maintain an even level of the medication in your body. **Potential side effects and complications:** Common side effects: Dizziness or lightheadedness. Fatigue or tiredness. Nausea or stomach upset. Increased urination. Cold hands or feet. Serious side effects: Severe allergic reactions (rash, itching/swelling, severe dizziness, trouble breathing). Signs of low blood pressure (extreme dizziness, fainting). Signs of electrolyte imbalance (dry mouth, increased thirst, muscle cramps, weakness, confusion, irregular heartbeat). Slow or irregular heartbeat. Shortness of breath, wheezing, or swelling of the ankles/feet. **Note:** Always follow your healthcare provider's instructions closely and report any unusual symptoms or side effects immediately. Bisoprolol can mask signs of low blood sugar in diabetic patients, so monitor your blood sugar levels closely. Regular follow-ups with your healthcare provider are important to monitor your condition and the effects of the medication. Avoid abrupt discontinuation of the medication without consulting your doctor, as it may worsen your condition.
**4**	Picture of: Ganaton®	The medication shown in the image is Ganaton 50 mg, which contains the active ingredient itopride hydrochloride. **What is it?** Itopride hydrochloride is a prokinetic agent, which means it helps to enhance gastrointestinal motility. It works by increasing the movement of the stomach and intestines, facilitating the movement of food and alleviating symptoms of digestive disorders. **What is it used for?** Ganaton is used to treat symptoms of: Functional dyspepsia (indigestion). Gastroesophageal reflux disease (GERD). Other gastrointestinal motility disorders. **How should you use it?** Dosage: Follow your doctor's prescription exactly. The usual dose is 50 mg taken three times daily before meals. Administration: Take the tablet orally with a glass of water. It is typically taken before meals. Consistency: Take it at the same times each day to maintain an even level of the medication in your body. **Potential side effects and complications:** Common side effects: Headache. Dizziness. Diarrhea or constipation. Abdominal pain. Serious side effects: Severe allergic reactions (rash, itching/swelling, severe dizziness, trouble breathing). Changes in heart rate. Tremors or other abnormal movements. **Note:** Always follow your healthcare provider's instructions closely and report any unusual symptoms or side effects immediately. Itopride can interact with other medications, so inform your doctor about all medications and supplements you are taking. Regular follow-ups with your healthcare provider are important to monitor your condition and the effects of the medication.

**5**	Picture of: Lanoxin®	The medication shown in the image is Lanoxin Paediatric/Geriatric Elixir, which contains the active ingredient digoxin at a concentration of 0.05 mg/mL. **What is it?** Digoxin is a medication derived from the foxglove plant and is used to treat various heart conditions. It belongs to a class of drugs called cardiac glycosides. **What is it used for?** Lanoxin is used to: Treat heart failure. Manage certain types of irregular heartbeats (arrhythmias), particularly atrial fibrillation. **How should you use it?** Dosage: Follow your doctor's prescription exactly. The dosage depends on the specific condition being treated, as well as the patient's age, weight, and kidney function. Administration: Use a proper measuring device to ensure the correct dose. Administer the elixir orally, and it can be given with or without food. Monitoring: Regular blood tests may be needed to ensure the correct digoxin levels in your blood and to adjust the dose if necessary. **Potential side effects and complications:** Common side effects: Nausea, vomiting, and loss of appetite. Diarrhea. Dizziness or lightheadedness. Fatigue and weakness. Serious side effects: Irregular heartbeats (arrhythmias), which can be life-threatening. Severe allergic reactions (rash, itching/swelling of the face, tongue, or throat, severe dizziness, trouble breathing). Digoxin toxicity, especially if used improperly or in higher-than-prescribed doses. Symptoms include confusion, blurred vision, seeing halos around lights, and severe stomach pain. **Note:** Always follow your healthcare provider's instructions closely and report any unusual symptoms or side effects immediately. Digoxin levels can be affected by other medications and certain medical conditions, so it is important to inform your healthcare provider about all medications you are taking and any other health conditions you have. Regular monitoring of blood levels and kidney function is often necessary when taking digoxin.
**6**	Picture of: Lantus® SoloStar®	The medication shown in the image is Lantus SoloStar, which contains the active ingredient insulin glargine. **What is it?** Lantus SoloStar is a long-acting insulin used to improve blood sugar control in people with diabetes mellitus. It is usually administered once daily. **What is it used for?** Lantus SoloStar is used to: Treat type 1 diabetes in adults and children. Treat type 2 diabetes in adults. **How should you use it?** Dosage: Follow your doctor's prescription. The dosage is individualized based on your blood glucose levels and medical condition. Administration: Inject Lantus SoloStar subcutaneously (under the skin), typically in the abdomen, thigh, or upper arm. Rotate injection sites to reduce the risk of lipodystrophy (changes in fat tissue) at the injection site. Timing: Lantus is usually taken once daily, at the same time each day. Preparation: Before using, inspect the insulin solution. It should be clear and colorless. Do not use it if it appears cloudy, thickened, or contains particles. **Potential side effects and complications:** Common side effects: Hypoglycemia (low blood sugar), which can cause symptoms like dizziness, sweating, confusion, and headache. Injection site reactions (redness, swelling, itching). Weight gain. Serious side effects: Severe hypoglycemia (confusion, seizures, loss of consciousness). Allergic reactions (rash, itching, difficulty breathing, swelling of the face, tongue, or throat). **Note:** Always follow your healthcare provider's instructions and regularly monitor your blood sugar levels. Report any unusual symptoms or side effects immediately. It's crucial to have a source of sugar (e.g., glucose tablets or gel) readily available in case of hypoglycemia.

**7**	Picture of: Lasix®	The medication shown in the image is Lasix 40 mg, which contains the active ingredient furosemide. **What is it?** Furosemide is a loop diuretic (water pill) that helps prevent your body from absorbing too much salt, allowing the salt to instead be passed in your urine. **What is it used for?** Lasix is used to: Treat fluid retention (edema) in people with congestive heart failure, liver disease, or a kidney disorder such as nephrotic syndrome. Treat high blood pressure (hypertension). **How should you use it?** Dosage: Follow your doctor's prescription exactly. The dosage will depend on your specific condition and response to treatment. Administration: Take it orally, usually once or twice daily. It can be taken with or without food. If taking it twice daily, take it in the morning and afternoon to avoid needing to urinate during the night. Monitoring: Regular monitoring of kidney function and electrolyte levels (e.g., potassium, sodium) is necessary while taking this medication. **Potential side effects and complications:** Common side effects: Increased urination. Dizziness or lightheadedness, especially when standing up quickly. Dehydration and electrolyte imbalances (low potassium, sodium, magnesium). Serious side effects: Severe dehydration (dry mouth, excessive thirst, muscle cramps, weakness). Hearing problems or ringing in the ears. Severe allergic reactions (rash, itching/swelling, severe dizziness, trouble breathing). Signs of kidney problems (change in the amount of urine). **Note:** Always follow your healthcare provider's instructions closely and report any unusual symptoms or side effects immediately. It's important to maintain adequate hydration while taking this medication. Regular check-ups and blood tests are necessary to monitor your progress and adjust the dose if needed. Avoid taking other medications or supplements without consulting your doctor, as many can interact with furosemide.
**8**	Picture of: Lyrica®	The medication shown in the image is Lyrica 75 mg, which contains the active ingredient pregabalin. **What is it?** Pregabalin is an anticonvulsant and neuropathic pain agent. It works by slowing down impulses in the brain that cause seizures and affects chemicals in the brain that send pain signals across the nervous system. **What is it used for?** Lyrica is used to treat: Neuropathic pain associated with diabetic peripheral neuropathy. Neuropathic pain associated with spinal cord injury. Post-herpetic neuralgia. Fibromyalgia. Partial onset seizures in adults (as an adjunctive therapy). **How should you use it?** Dosage: Follow your doctor's prescription exactly. The dosage will depend on the specific condition being treated and your response to the medication. Administration: Take the capsule orally, usually two or three times daily. It can be taken with or without food. Consistency: Take the medication at the same times each day to maintain an even level of the drug in your body. **Potential side effects and complications:** Common side effects: Dizziness. Drowsiness. Dry mouth. Swelling in the hands and feet. Blurred vision. Weight gain. Serious side effects: Severe allergic reactions (rash, itching/swelling, severe dizziness, trouble breathing). Suicidal thoughts or behavior. Angioedema (swelling of the face, mouth, or throat). Muscle pain, tenderness, or weakness (especially if accompanied by fever or malaise). Signs of kidney problems (change in the amount of urine). **Note:** Always follow your healthcare provider's instructions closely and report any unusual symptoms or side effects immediately. Pregabalin can interact with other medications, so inform your doctor about all medications and supplements you are taking. Do not suddenly stop taking this medication without consulting your healthcare provider, as it may cause withdrawal symptoms. Regular follow-ups with your healthcare provider are important to monitor your condition and the effects of the medication.

**9**	Picture of: Nexium®	The medication shown in the image is Nexium 40 mg, which contains the active ingredient esomeprazole. **What is it?** Esomeprazole is a proton pump inhibitor (PPI) that decreases the amount of acid produced in the stomach. **What is it used for?** Nexium is used to: Treat gastroesophageal reflux disease (GERD). Heal erosive esophagitis (damage to the esophagus from stomach acid). Prevent gastric ulcers caused by infection with Helicobacter pylori or the use of nonsteroidal anti-inflammatory drugs (NSAIDs). Treat conditions involving excessive stomach acid such as Zollinger-Ellison syndrome. **How should you use it?** Dosage: Follow your doctor's prescription exactly. The typical dose for treating GERD is 20-40 mg once daily for 4 to 8 weeks. Administration: Take the tablet at least one hour before a meal. Swallow it whole with a glass of water; do not crush or chew the tablet. Consistency: Take the medication at the same time each day to maintain an even level of the drug in your body. **Potential side effects and complications:** Common side effects: Headache. Diarrhea. Nausea. Flatulence. Stomach pain. Serious side effects: Severe allergic reactions (rash, itching, swelling of the face, tongue, or throat, severe dizziness, trouble breathing). Low magnesium levels (symptoms may include dizziness, confusion, fast or uneven heart rate, muscle spasms, cough or choking feeling). Clostridium difficile-associated diarrhea (persistent diarrhea, abdominal pain, fever). Vitamin B-12 deficiency if used long-term. **Note:** Always follow your healthcare provider's instructions closely and report any unusual symptoms or side effects immediately. Long-term use of esomeprazole can lead to an increased risk of bone fractures, low magnesium levels, and vitamin B-12 deficiency, so regular follow-ups with your healthcare provider are important. Avoid taking other medications or supplements without consulting your doctor, as many can interact with PPIs.
**10**	Picture of: Omnic Ocas®	The medication shown in the image is Omnic Ocas 0.4, which contains the active ingredient tamsulosin hydrochloride. **What is it?** Tamsulosin hydrochloride is an alpha-blocker that helps relax the muscles in the prostate and bladder neck, making it easier to urinate. **What is it used for?** Omnic Ocas is used to treat the symptoms of an enlarged prostate (benign prostatic hyperplasia - BPH). It helps relieve symptoms such as difficulty in beginning the flow of urine, weak stream, and the need to urinate frequently or urgently (including during the middle of the night). **How should you use it?** Dosage: Follow your doctor's prescription exactly. The usual dose is one tablet (0.4 mg) taken once daily. Administration: Take the tablet orally, after the same meal each day. Swallow it whole without crushing, chewing, or opening it. Consistency: It's important to take it at the same time each day to maintain an even level of the medication in your body. **Potential side effects and complications:** Common side effects: Dizziness, particularly when standing up from a sitting or lying position (orthostatic hypotension). Ejaculation problems. Runny or stuffy nose. Headache. Serious side effects: Severe dizziness or fainting. Priapism (painful or prolonged erection). Severe allergic reactions (rash, itching, swelling of the face, tongue, or throat, severe dizziness, trouble breathing). **Note:** Always follow your healthcare provider's instructions and report any unusual symptoms or side effects immediately. If you are planning to have cataract or glaucoma surgery, inform your surgeon that you are using tamsulosin, as it can affect your pupils during surgery.

**11**	Picture of: Orfarin®	The medication shown in the image is Orfarin 3 mg, which contains the active ingredient Warfarin sodium. **What is it?** Warfarin is an anticoagulant (blood thinner). It reduces the formation of blood clots by blocking the formation of certain clotting factors. **What is it used for?** Warfarin is used to: Prevent blood clots in veins or arteries, which can reduce the risk of stroke, heart attack, or other serious conditions. Treat or prevent venous thrombosis (blood clots in veins) and pulmonary embolism (blood clots in lungs). Manage patients with atrial fibrillation, heart valve replacement, or other conditions where blood clot risk is increased. **How should you use it?** Dosage: Follow your doctor's prescription exactly. The dosage of warfarin is individualized based on your blood test results (INR - International Normalized Ratio). Administration: Take it orally, with or without food, preferably at the same time each day. Monitoring: Regular blood tests (INR) are necessary to ensure the medication is working effectively and safely. Your doctor will adjust your dose based on these tests. Diet: Maintain a consistent diet, especially regarding foods high in vitamin K (e.g., leafy greens), as they can affect how warfarin works. Avoid: Inform your doctor about all medications and supplements you are taking, as many can interact with warfarin. **Potential side effects and complications:** Common side effects: Bleeding, including gums, nosebleeds, heavier menstrual periods, or bleeding from cuts that take longer to stop. Bruising more easily. Serious side effects: Severe bleeding (unusual pain, swelling, or discomfort; prolonged bleeding from cuts; frequent nosebleeds; unusual bruising; red or dark brown urine; red or tar-black stools; coughing up or vomiting blood). Signs of a stroke (sudden severe headache, confusion, problems with vision, speech, or balance). Signs of a blood clot (swelling, warmth, or pain in a leg or arm; chest pain; difficulty breathing). **Note:** Always follow your healthcare provider's instructions and report any unusual symptoms or side effects immediately.
**12**	Picture of: Primolut® Depot	The medication shown in the image is Primolut Depot, which contains the active ingredient hydroxyprogesterone caproate. **What is it?** Hydroxyprogesterone caproate is a synthetic form of the hormone progesterone. It is used to prevent preterm birth in women with a history of preterm delivery. **What is it used for?** Primolut Depot is primarily used for: Reducing the risk of preterm birth in women who have had a previous preterm delivery. Treating certain menstrual disorders and other conditions as determined by your doctor. **How should you use it?** Dosage: Follow your doctor's prescription exactly. The dosage and schedule will depend on your specific condition and medical history. Administration: This medication is given as an intramuscular injection (injected into a muscle) by a healthcare professional. Frequency: Typically administered once a week, starting during the second trimester of pregnancy and continuing until 37 weeks of gestation or as directed by your healthcare provider. **Potential side effects and complications:** Common side effects: Pain, swelling, or irritation at the injection site. Nausea. Headache. Itching or rash. Serious side effects: Allergic reactions (hives, difficulty breathing, swelling of the face, lips, tongue, or throat). Blood clots (pain, swelling, warmth in one leg, sudden chest pain, shortness of breath). Signs of liver problems (nausea, upper stomach pain, itching, tired feeling, loss of appetite, dark urine, clay-colored stools, jaundice). Depression or mood changes. **Note:** Always follow your healthcare provider's instructions and report any unusual symptoms or side effects immediately. Keep all scheduled appointments to monitor your condition and the effects of the medication.

**13**	Picture of: Procto-glyvenol®	The medication shown in the image is Procto-Glyvenol, which contains the active ingredients tribenoside and lidocaine. **What is it?** Procto-Glyvenol is a combination medication used to treat hemorrhoids. Tribenoside has anti-inflammatory properties, while lidocaine is a local anesthetic that helps to relieve pain and itching. **What is it used for?** Procto-Glyvenol is used for the local treatment of: Internal and external hemorrhoids. **How should you use it?** Dosage: Follow your doctor's prescription exactly. The typical dose is one suppository administered once or twice daily. Administration: Wash your hands before and after using the suppository. If the suppository is too soft to insert, chill it in the refrigerator for a few minutes or run cold water over it before unwrapping. Lie down on your side and insert the suppository into the rectum pointed end first. Push it in about 1 inch to 1.5 inches (2.5 to 3.75 cm) in adults. Frequency: Use as directed by your healthcare provider, usually once in the morning and once in the evening initially, then once daily as symptoms improve. **Potential side effects and complications:** Common side effects: Localized burning sensation. Itching or redness at the application site. Serious side effects: Severe allergic reactions (rash, itching/swelling, severe dizziness, trouble breathing). **Note:** Always follow your healthcare provider's instructions closely and report any unusual symptoms or side effects immediately. Do not use this medication for longer than prescribed, and avoid using it if you have known allergies to any of its ingredients. If symptoms persist or worsen, consult your healthcare provider.
**14**	Picture of: Rennie®	The medication shown in the image is Rennie Peppermint, which contains the active ingredients calcium carbonate and magnesium carbonate. **What is it?** Rennie is an antacid used to neutralize stomach acid, providing relief from indigestion and heartburn. The peppermint flavor helps to freshen breath and improve the taste. **What is it used for?** Rennie is used to: Relieve symptoms of indigestion (dyspepsia). Provide relief from heartburn. Alleviate the discomfort caused by excess stomach acid. **How should you use it?** Dosage: Follow the instructions on the package or as directed by your healthcare provider. The usual dose is one or two tablets as required. Administration: Chew or suck the tablets. Do not swallow them whole. Frequency: Use as needed, but do not exceed the maximum recommended dose, which is usually specified on the packaging. **Potential side effects and complications:** Common side effects: Constipation. Diarrhea. Flatulence. Serious side effects: Hypercalcemia (high levels of calcium in the blood), which can cause symptoms like nausea, vomiting, confusion, and weakness. Hypomagnesemia (low levels of magnesium in the blood), especially if used in large amounts or for prolonged periods. **Note:** Always follow the recommended dosage and consult your healthcare provider if symptoms persist or worsen. Antacids can interact with other medications, so inform your doctor about all medications you are taking. Avoid using Rennie for prolonged periods without medical advice, as it is intended for temporary relief.

**15**	Picture of: Seretide® MDI	The medication shown in the image is Seretide MDI 125/25, which contains the active ingredients fluticasone propionate and salmeterol xinafoate. **What is it?** Seretide is a combination inhaler that contains fluticasone propionate, a corticosteroid that reduces inflammation, and salmeterol xinafoate, a long-acting beta-agonist (LABA) that helps to relax muscles in the airways to improve breathing. **What is it used for?** Seretide is used to: Manage asthma in patients who need both corticosteroid and long-acting beta-agonist medications. Treat chronic obstructive pulmonary disease (COPD), including chronic bronchitis and emphysema, to reduce flare-ups and improve breathing. **How should you use it?** Dosage: Follow your doctor's prescription exactly. The typical dose for adults is two inhalations twice daily, but the exact dosage will depend on your specific condition. Administration: Shake the inhaler well before each use. Breathe out fully. Place the mouthpiece between your lips and close your lips around it. Breathe in deeply and press down on the inhaler to release a dose. Hold your breath for about 10 seconds, then breathe out slowly. If you need more than one inhalation, wait about 30 seconds before repeating the steps. Frequency: Use it at the same times each day, typically once in the morning and once in the evening. **Potential side effects and complications:** Common side effects: Hoarseness or throat irritation. Oral thrush (fungal infection in the mouth). Headache. Nausea. Muscle and bone pain. Serious side effects: Severe allergic reactions (rash, itching/swelling, severe dizziness, trouble breathing). High blood pressure. Fast or irregular heartbeat. Chest pain. Signs of infection (fever, chills, persistent sore throat). **Note:** Always follow your healthcare provider's instructions closely and report any unusual symptoms or side effects immediately. Rinse your mouth with water after each use to reduce the risk of oral thrush. Do not use Seretide to treat an acute asthma attack. Ensure regular follow-ups with your healthcare provider to monitor your condition and the effects of the medication.
**16**	Picture of: Spiriva®	The medication shown in the image is Spiriva 18 mcg, which contains the active ingredient tiotropium bromide. **What is it?** Tiotropium bromide is a long-acting anticholinergic bronchodilator. It helps to relax the muscles around the airways so that they open up and you can breathe more easily. **What is it used for?** Spiriva is used to: Treat chronic obstructive pulmonary disease (COPD), including chronic bronchitis and emphysema. Prevent bronchospasm (narrowing of the airways in the lungs) in people with COPD. **How should you use it?** Dosage: Follow your doctor's prescription exactly. The usual dose is one capsule inhaled once daily. Administration: Use the HandiHaler device that comes with the medication. Do not swallow the capsules. Insert one capsule into the HandiHaler device. Press the button on the device to pierce the capsule. Breathe in the medication through your mouth using the device. Frequency: Use the inhaler at the same time each day to maintain an even level of the medication in your body. **Potential side effects and complications:** Common side effects: Dry mouth. Constipation. Blurred vision. Cough. Sore throat. Serious side effects: Severe allergic reactions (rash, itching, swelling of the face, tongue, or throat, severe dizziness, trouble breathing). Glaucoma (eye pain, blurred vision, halos around lights). Urinary retention (difficulty urinating, pain when urinating). **Note:** Always follow your healthcare provider's instructions and report any unusual symptoms or side effects immediately. Keep all scheduled appointments to monitor your condition and the effects of the medication. Use the HandiHaler device as instructed and ensure you do not swallow the capsules.

**17**	Picture of: Symbicort® Turbuhaler®	The medication shown in the image is Symbicort Turbuhaler 320/9 µg/dose, which contains the active ingredients budesonide and formoterol. **What is it?** Symbicort is a combination inhaler that contains budesonide, a corticosteroid that reduces inflammation, and formoterol, a long-acting beta-agonist (LABA) that helps to relax muscles in the airways to improve breathing. **What is it used for?** Symbicort is used to: Manage asthma in patients who need both corticosteroid and long-acting beta-agonist medications. Treat chronic obstructive pulmonary disease (COPD), including chronic bronchitis and emphysema, to reduce flare-ups and improve breathing. **How should you use it?** Dosage: Follow your doctor's prescription exactly. The typical dose for adults is one inhalation twice daily, but the exact dosage will depend on your specific condition. Administration: Remove the cap from the Turbuhaler. Hold the inhaler upright and twist the grip to load a dose. Breathe out fully (away from the inhaler). Place the mouthpiece between your lips and inhale deeply and forcefully. Hold your breath for 10 seconds, then breathe out slowly. Replace the cap after use. Frequency: Use it at the same times each day, typically once in the morning and once in the evening. **Potential side effects and complications:** Common side effects: Hoarseness or throat irritation. Oral thrush (fungal infection in the mouth). Headache. Nausea. Cough. Serious side effects: Severe allergic reactions (rash, itching/swelling, severe dizziness, trouble breathing). High blood pressure. Fast or irregular heartbeat. Chest pain. Signs of infection (fever, chills, persistent sore throat). **Note:** Always follow your healthcare provider's instructions closely and report any unusual symptoms or side effects immediately. Rinse your mouth with water after each use to reduce the risk of oral thrush. Do not use Symbicort to treat an acute asthma attack. Ensure regular follow-ups with your healthcare provider to monitor your condition and the effects of the medication.
**18**	Picture of: Tobrex®	The medication shown in the image is Tobrex (tobramycin) Eye Ointment 3.5 g, which contains the active ingredient tobramycin. **What is it?** Tobramycin is an antibiotic that belongs to the aminoglycoside class. It is used to treat bacterial infections by inhibiting bacterial protein synthesis. **What is it used for?** Tobrex Eye Ointment is used to treat: Bacterial infections of the eyes such as conjunctivitis (pink eye), keratitis, and blepharitis. Other bacterial infections of the eye as prescribed by your healthcare provider. **How should you use it?** Dosage: Follow your doctor's prescription exactly. The typical application is a small amount (approximately 1/2 inch ribbon) of ointment in the affected eye(s) 2-3 times daily. Administration: Wash your hands thoroughly before using the eye ointment. Tilt your head back and gently pull down the lower eyelid to create a small pocket. Squeeze a small amount of ointment into the pocket. Close your eyes for a few moments and gently roll your eyes to distribute the ointment. Avoid touching the tip of the tube to any surface, including your eye or hands, to prevent contamination. Frequency: Use as directed by your healthcare provider, typically 2-3 times daily for the prescribed duration. **Potential side effects and complications:** Common side effects: Temporary blurred vision. Mild eye irritation or redness. Stinging or burning sensation after application. Serious side effects: Severe allergic reactions (rash, itching/swelling, severe dizziness, trouble breathing). Eye pain or swelling. Signs of a new eye infection (discharge, swelling, redness). **Note:** Always follow your healthcare provider's instructions closely and report any unusual symptoms or side effects immediately. Do not use Tobrex for longer than prescribed, as overuse of antibiotics can lead to secondary infections. If you wear contact lenses, remove them before applying the ointment and wait at least 15 minutes before reinserting them. Ensure regular follow-ups with your healthcare provider to monitor your condition and the effects of the medication.

**19**	Picture of: Vibrocil®	The medication shown in the image is Vibrocil Nasal Gel, which contains the active ingredients dimethindene maleate and phenylephrine hydrochloride. **What is it?** Dimethindene maleate is an antihistamine that helps to reduce allergy symptoms. Phenylephrine hydrochloride is a decongestant that narrows the blood vessels in the nasal passages, reducing swelling and congestion. **What is it used for?** Vibrocil Nasal Gel is used to treat: Nasal congestion due to colds, allergies (such as hay fever), and sinusitis. Runny nose and sneezing due to allergic reactions. **How should you use it?** Dosage: Follow your doctor's prescription or the instructions on the package. Typically, the gel is applied in each nostril 3-4 times daily. Administration: Blow your nose gently before using the gel. Apply a small amount of gel into each nostril. Gently squeeze the sides of your nose together to spread the gel evenly. Avoid touching the tip of the tube to any surface to prevent contamination. Frequency: Use as directed, typically up to 3-4 times daily. **Potential side effects and complications:** Common side effects: Mild burning or stinging sensation in the nose. Dryness in the nose. Serious side effects: Severe allergic reactions (rash, itching/swelling, severe dizziness, trouble breathing). High blood pressure (headache, blurred vision, chest pain, shortness of breath). Palpitations or fast/irregular heartbeat. **Note:** Always follow your healthcare provider's instructions closely and report any unusual symptoms or side effects immediately. Do not use Vibrocil for more than one week continuously without medical advice, as prolonged use may lead to rebound congestion. If symptoms persist or worsen, consult your healthcare provider. Avoid using other decongestants concurrently unless advised by your doctor.
**20**	Picture of: Voltfast®	The medication shown in the image is Voltfast 50 mg, which contains the active ingredient diclofenac potassium. **What is it?** Diclofenac potassium is a nonsteroidal anti-inflammatory drug (NSAID) that is used to reduce inflammation and pain in the body. **What is it used for?** Voltfast is used to treat: Pain and inflammation from conditions such as arthritis. Acute musculoskeletal pain. Dysmenorrhea (menstrual pain). Other conditions requiring short-term treatment of moderate pain and inflammation. **How should you use it?** Dosage: Follow your doctor's prescription exactly. The typical dose for adults is 50 mg taken 2-3 times daily, but the exact dosage and frequency can vary based on the specific condition being treated. Administration: Dissolve the contents of one sachet in a glass of water and drink it immediately. It is best taken with food or after meals to minimize gastrointestinal discomfort. Frequency: Use as directed by your healthcare provider, usually not exceeding 150 mg per day. **Potential side effects and complications:** Common side effects: Stomach pain, nausea, or vomiting. Diarrhea or constipation. Headache or dizziness. Indigestion or heartburn. Serious side effects: Severe allergic reactions (rash, itching/swelling, severe dizziness, trouble breathing). Signs of stomach bleeding (bloody or black tarry stools, coughing up blood or vomit that looks like coffee grounds). Liver problems (yellowing of the skin or eyes, dark urine, severe stomach pain). Kidney problems (change in the amount of urine). Heart attack or stroke symptoms (chest pain, shortness of breath, weakness on one side of the body, slurred speech). **Note:** Always follow your healthcare provider's instructions closely and report any unusual symptoms or side effects immediately. NSAIDs like diclofenac can increase the risk of serious cardiovascular thrombotic events, myocardial infarction, and stroke, which can be fatal. They can also cause gastrointestinal adverse events including bleeding, ulceration, and perforation of the stomach or intestines, which can be fatal. Regular follow-ups with your healthcare provider are important to monitor your condition and the effects of the medication. Avoid taking other NSAIDs concurrently unless advised by your doctor.

Supplementary Table S2

No.	Input (blurred medication picture)	Response (output)
**1**	"I will show you a picture of a medication I was prescribed, and I want you to tell me what is it, what is it used for, how should I use it, and if there is any potential side effect or complication I should be aware of"<br><br>Picture of: Augmentin® (moderate blurring)	The medication in the picture is Augmentin. Augmentin is a combination antibiotic that contains two active ingredients: amoxicillin and clavulanate potassium. Here is some information about this medication: **What is it?** Amoxicillin is a penicillin-type antibiotic that helps stop the growth of bacteria. Clavulanate potassium is a beta-lactamase inhibitor that helps prevent certain bacteria from becoming resistant to amoxicillin. **What is it used for?** Augmentin is used to treat a variety of bacterial infections, including: Respiratory infections (e.g., sinusitis, pneumonia). Ear infections. Urinary tract infections. Skin infections. **How should you use it?** Dosage: Follow the dosage instructions provided by your healthcare provider. It is usually taken every 12 hours (twice daily) or every 8 hours (three times daily), depending on the specific formulation and the infection being treated. Administration: It is best taken at the start of a meal to reduce stomach upset. Swallow the tablet whole with a glass of water. Complete the course: It is essential to finish the entire prescribed course, even if you start feeling better, to ensure that the infection is fully treated and to prevent the development of antibiotic-resistant bacteria. **Potential side effects or complications:** Common side effects: Nausea, vomiting, diarrhea, and skin rash. Serious side effects: Severe allergic reactions (e.g., rash, itching, swelling, severe dizziness, trouble breathing), liver problems (e.g., dark urine, persistent nausea/vomiting, stomach/abdominal pain, yellowing eyes/skin), and severe intestinal condition (Clostridium difficile-associated diarrhea). Complications: If you experience severe side effects or signs of an allergic reaction, seek medical attention immediately. **Additional Notes:** Drug Interactions: Inform your doctor about any other medications you are taking, as Augmentin can interact with other drugs. Allergies: Make sure your healthcare provider is aware if you are allergic to penicillins or cephalosporins. If you have any specific concerns or additional questions about your medication, it is best to consult your healthcare provider or pharmacist.
**2**	Picture of: Seretide® MDI (High blurring)	The medication in the picture appears to be Seretide Evohaler. Seretide is a brand name for a combination of two active ingredients: fluticasone propionate and salmeterol xinafoate. **What is it?** Fluticasone propionate: A corticosteroid that helps to reduce inflammation in the lungs. Salmeterol xinafoate: A long-acting beta-agonist that helps to open up the airways in the lungs. **What is it used for?** Seretide Evohaler is used to manage and prevent symptoms of asthma and chronic obstructive pulmonary disease (COPD), including: Shortness of breath. Wheezing. Chronic cough. **How should you use it?** Dosage: Follow the dosage instructions provided by your healthcare provider. Typically, it is used twice a day (morning and evening). Administration: Shake the inhaler well before each use. Breathe out fully, place the mouthpiece in your mouth, and close your lips around it. As you start to breathe in slowly, press down on the inhaler to release the medication. Continue to breathe in slowly and deeply. Hold your breath for about 10 seconds, then breathe out slowly. Rinse your mouth with water after using the inhaler to reduce the risk of oral thrush (a common side effect). **Potential side effects or complications:** Common side effects: Hoarseness, throat irritation, headache, and increased heart rate. Serious side effects: Allergic reactions (e.g., rash, itching, swelling, severe dizziness, trouble breathing), worsening of breathing problems, and signs of infection (e.g., fever, persistent sore throat). Complications: Long-term use of corticosteroids can lead to bone loss (osteoporosis), eye problems (e.g., cataracts, glaucoma), and adrenal suppression. **Additional Notes:** Inhaler technique: Proper inhaler technique is crucial for the medication to be effective. If you have any difficulty using the inhaler, ask your healthcare provider for advice or a demonstration. Monitoring: Regular follow-up with your healthcare provider is important to monitor your response to the medication and adjust the dosage if necessary. If you have any specific concerns or additional questions about your medication, it is best to consult your healthcare provider or pharmacist.

**3**	Picture of: Crestor® (moderate blurring)	The medication in the image is Crestor, which contains the active ingredient rosuvastatin. **What is Crestor (Rosuvastatin)?** Crestor (rosuvastatin) is a statin medication used to lower cholesterol and triglycerides in the blood. It belongs to a class of drugs called HMG CoA reductase inhibitors (statins). **Uses:** Lowering Cholesterol: It helps reduce levels of "bad" cholesterol (low-density lipoprotein, or LDL) and triglycerides in the blood, while increasing levels of "good" cholesterol (high-density lipoprotein, or HDL). Preventing Cardiovascular Disease: Crestor is used to lower the risk of stroke, heart attack, and other heart complications in people with diabetes, coronary heart disease, or other risk factors. **How to Use Crestor:** Dosage: Follow your doctor's instructions regarding the dosage. Typically, it is taken once a day. Administration: Take Crestor with or without food. Swallow the tablet whole with a glass of water. Timing: Try to take it at the same time each day to maintain an even level in your blood. **Potential Side Effects:** Common Side Effects: Headache, muscle pain, abdominal pain, weakness, nausea. Serious Side Effects: Unexplained muscle pain, tenderness, or weakness (may indicate muscle breakdown). Liver problems (symptoms may include yellowing of the skin or eyes, dark urine, severe stomach pain, or nausea). Kidney problems (symptoms may include changes in the amount of urine) Allergic Reactions: Rash. Itching or swelling, especially of the face, tongue, or throat. Severe dizziness. Trouble breathing. Precautions: Medical History: Inform your doctor if you have any history of liver disease, kidney disease, or alcohol use. Pregnancy and Breastfeeding: Rosuvastatin can harm an unborn baby or pass into breast milk. It should not be used during pregnancy or breastfeeding. Interactions: Tell your doctor about all other medications you are taking as Crestor can interact with other drugs. Always follow the guidance of your healthcare provider regarding the use of any medication. If you experience any severe side effects or signs of an allergic reaction, seek medical attention immediately.
**4**	Picture of: Glucophage® (High blurring)	The medication in the image appears to be Glucophage, which contains the active ingredient metformin. **What is Glucophage (Metformin)?** Glucophage (metformin) is an oral diabetes medicine that helps control blood sugar levels. It is commonly used in the treatment of type 2 diabetes. **Uses:** Type 2 Diabetes: Metformin is used to improve blood sugar control in people with type 2 diabetes. Polycystic Ovary Syndrome (PCOS): It is sometimes used off-label to treat polycystic ovary syndrome. **How to Use Glucophage:** Dosage: Follow your doctor's instructions regarding the dosage. Typically, it is taken once or twice a day. Administration: Take Glucophage with meals to reduce gastrointestinal side effects. Swallow the tablet whole with a glass of water. Timing: It is usually taken with breakfast or the first main meal of the day. **Potential Side Effects:** Common Side Effects: Nausea, vomiting, stomach upset, diarrhea, weakness, metallic taste in the mouth. Serious Side Effects: Lactic acidosis (rare but serious; symptoms include muscle pain, feeling cold, trouble breathing, dizziness, and irregular heart rate). Vitamin B12 deficiency (symptoms may include fatigue, weakness, and pale skin). Allergic Reactions: Rash. Itching or swelling, especially of the face, tongue, or throat. Severe dizziness. Trouble breathing. Precautions: Medical History: Inform your doctor if you have any history of kidney disease, liver disease, heart disease, or alcohol use. Pregnancy and Breastfeeding: Metformin can be used during pregnancy under a doctor's guidance. It is also considered safe during breastfeeding, but always consult your healthcare provider. Interactions: Tell your doctor about all other medications you are taking as Glucophage can interact with other drugs. Monitoring: Regularly monitor blood sugar levels as directed by your doctor. Periodic blood tests may be required to check kidney function and vitamin B12 levels. Always follow the guidance of your healthcare provider regarding the use of any medication. If you experience any severe side effects or signs of an allergic reaction, seek medical attention immediately.

Supplementary Table S3

No.	Medication (input)	Generated Image (output) – The brand names were masked, by the authors, in respect of copyrights
1	"I was prescribed a medication, I will show it to you in a picture, can you generate an image, or series of images, that shows a step-by-step guide on how to use it?" <br>Picture of: Lantus® SoloStar®	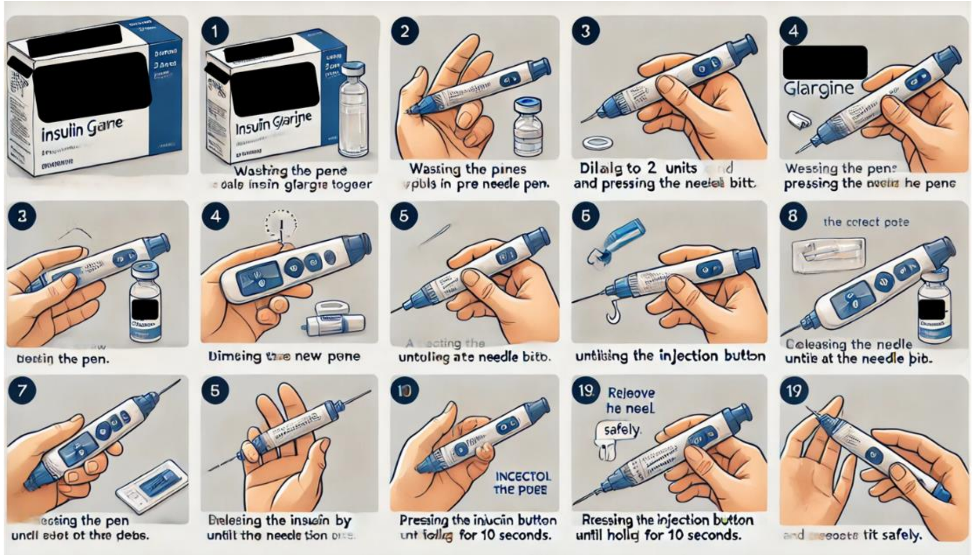
2	"I want you to do the same for this drug" <br>Picture of: Seretide® MDI	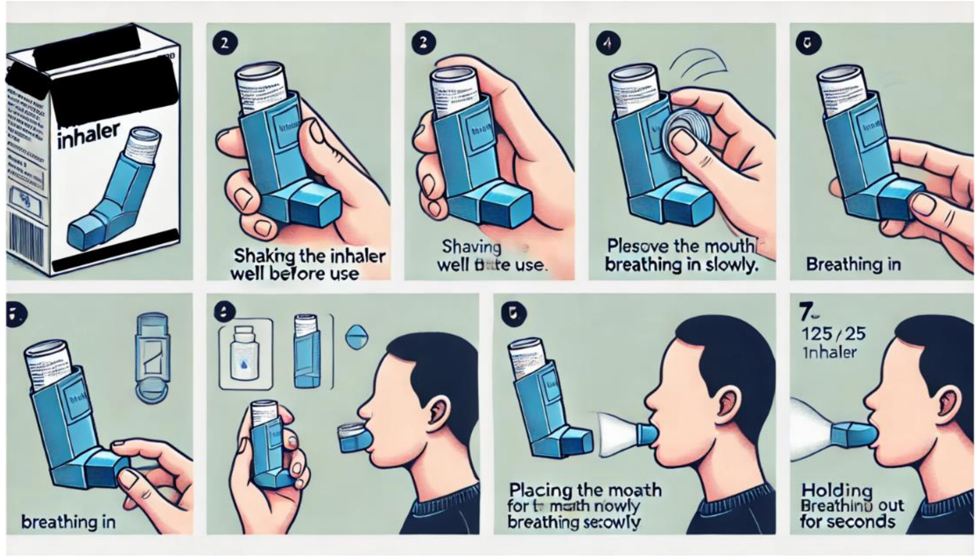

**3**	“I want you to do the same for this drug”<br><br>Picture of: Tobrex®	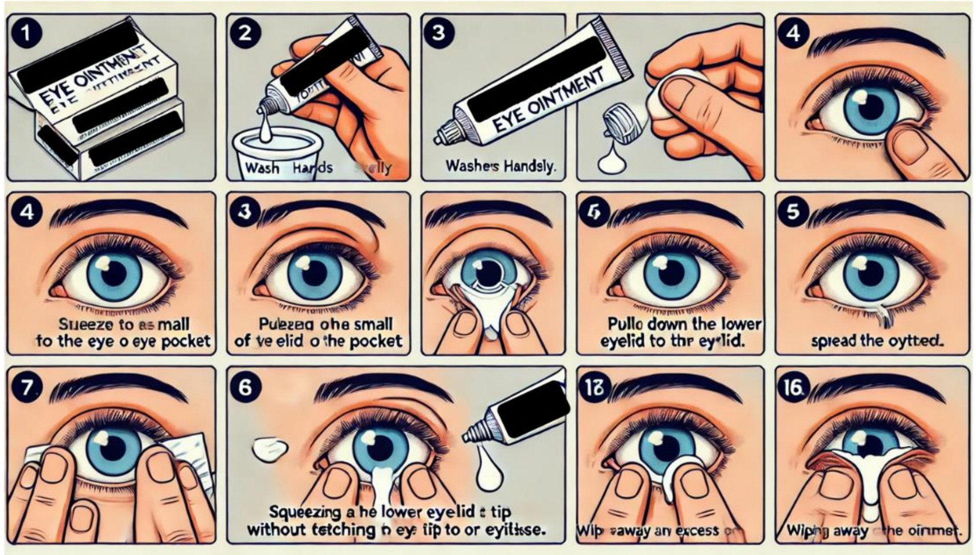
**4**	“I want you to do the same for this drug”<br><br>Picture of: Vibrocil®	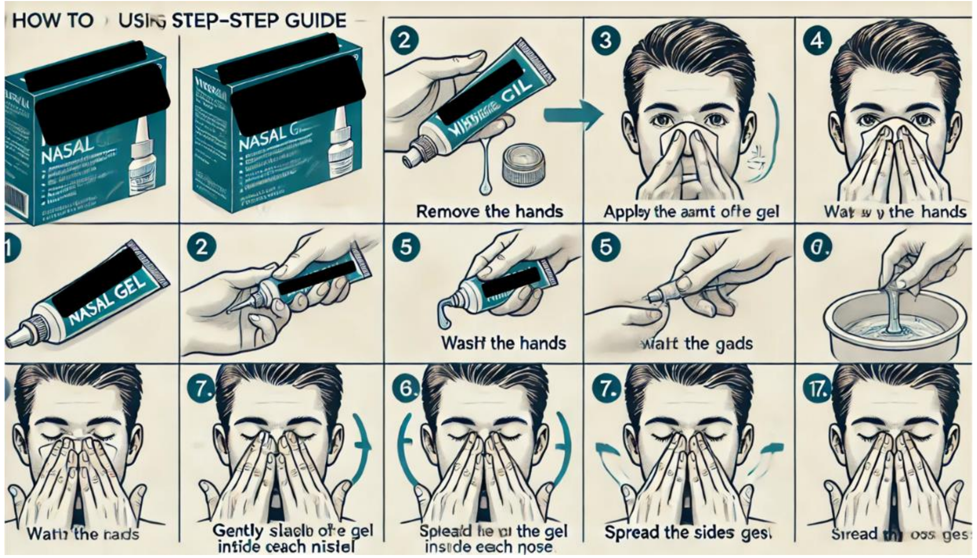
